# 

**Published:** 1911-03-11

**Authors:** 


Telegraphic Address: Ospedale, London.

THE MODERN MEDICAL AND INSTITUTIONAL JOURNAL.

Telephone Number: 2734 Gerrard.

NEW SERIES. No. 214, Vol. VIII. No. 1286, Vol. XLIX.

SATURDAY, MARCH 11TH, 1911.

PRICE THREEPENCE

